# Defect Engineering on Carbon-Based Catalysts for Electrocatalytic CO_2_ Reduction

**DOI:** 10.1007/s40820-020-00538-7

**Published:** 2020-10-27

**Authors:** Dongping Xue, Huicong Xia, Wenfu Yan, Jianan Zhang, Shichun Mu

**Affiliations:** 1 College of Materials Science and Engineering, Zhengzhou University, Zhengzhou, 450001 People’s Republic of China; 2 State Key Laboratory of Inorganic Synthesis and Preparative Chemistry, and College of Chemistry, Jilin University, 2699 Qianjin Street, Changchun, 130012 People’s Republic of China; 3 State Key Laboratory of Advanced Technology for Materials Synthesis and Processing, Wuhan University of Technology, Wuhan, 430070 People’s Republic of China

**Keywords:** Electrocatalytic CO_2_ reduction, Carbon-based nanomaterials, Intrinsic defects, Heteroatom doping defects, Metal atomic sites

## Abstract

Electrocatalytic carbon dioxide (CO_2_) reduction (ECR) has become one of the main methods to close the broken carbon cycle and temporarily store renewable energy, but there are still some problems such as poor stability, low activity, and selectivity. While the most promising strategy to improve ECR activity is to develop electrocatalysts with low cost, high activity, and long-term stability. Recently, defective carbon-based nanomaterials have attracted extensive attention due to the unbalanced electron distribution and electronic structural distortion caused by the defects on the carbon materials. Here, the present review mainly summarizes the latest research progress of the construction of the diverse types of defects (intrinsic carbon defects, heteroatom doping defects, metal atomic sites, and edges detects) for carbon materials in ECR, and unveil the structure–activity relationship and its catalytic mechanism. The current challenges and opportunities faced by high-performance carbon materials in ECR are discussed, as well as possible future solutions. It can be believed that this review can provide some inspiration for the future of development of high-performance ECR catalysts.

## Highlights

- The main construction methods of carbon-based nanomaterials (CBN) with different defects are systematically introduced.
- The structure–activity relationship of defective carbon-based catalysts in electrocatalytic carbon dioxide reduction (ECR) reaction is mainly reviewed.
- Challenges and opportunities of high-performance defective CBN in ECR and the possible solutions in the future are discussed.

## Graphical abstract

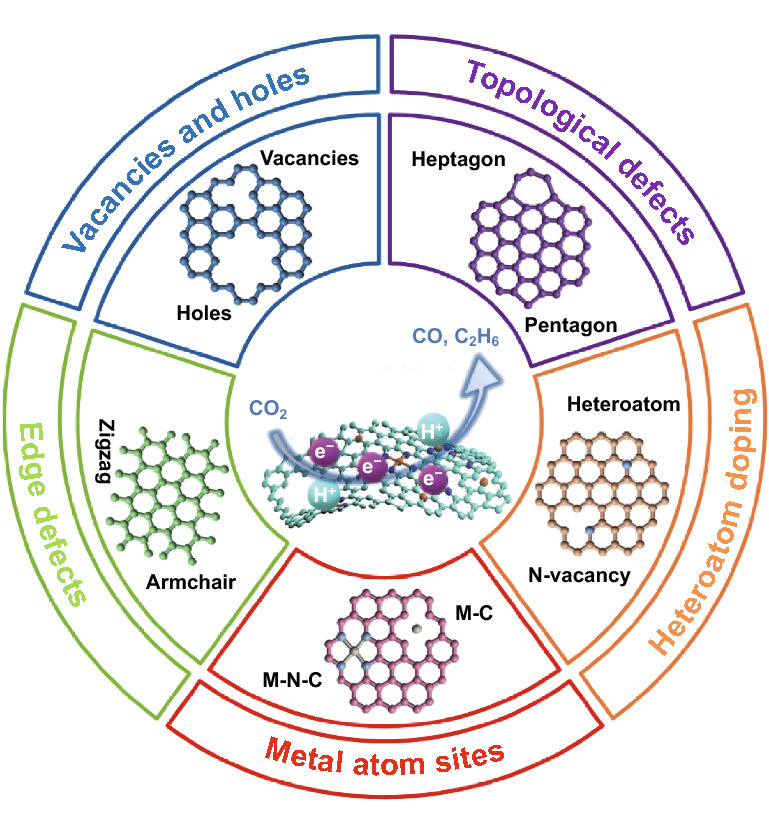

## Introduction

With the progress of human society, the continuous and rapid growth of energy demand has led to the increasing consumption of fossil fuels. Excessive carbon dioxide (CO_2_) generated by combustion is discharged into the atmosphere, which seriously disrupts the original normal carbon cycle of nature, causing global warming, and then causes a series of serious environmental problems [1, 2], such as sea level rise, land desertification, and climate abnormality. Therefore, the capture and conversion of CO_2_ into fuel or chemical raw materials with high added value has become one of the hot spots of scientific research because it can provide solutions to carbon emissions and energy crisis at the same time [3–8]. In the past few decades, various technologies such as biochemical, electronic, photochemical, radiochemical, and thermochemical have been developed to reduce CO_2_ [9–12]. Among them, electrocatalytic CO_2_ reduction (ECR) is an effective way to end man-made carbon cycle and store renewable energy by virtue of its simple experimental equipment, mild reaction conditions and the use of renewable energy to provide power [13–15]. Moreover, the ECR technology uses renewable energy sources such as solar energy, wind energy, and tidal energy to effectively reduce CO_2_ to important fuels and chemical raw materials. For example, ethanol with high energy density is a fuel with high octane number. Its market price is high and the global demand is continuous. The current global market for ethanol has reached US $75 billion and is estimated to reach US $105 billion by the end of 2022 [16]. Carbon monoxide (CO) as an important chemical raw material, can be used to produce various chemical products, including phosgene, methanol, and fatty aldehydes. It can also be directly used to produce liquid hydrocarbons in gasoline and diesel through Fischer–Tropsch synthesis. However, most of CO is produced by coal gasification or steam methane reforming, both of which rely on fossil fuels as raw materials. Therefore, the ECR technology provides a renewable way for the synthesis of CO [17].

Although ECR technology has great advantages and potential, it still faces some challenges, which must be solved before commercialization. Linear CO_2_ molecules has relatively thermodynamic stability and chemical inertness, which slows down the kinetics of the reduction reaction. The initial C=O double bond energy of CO_2_ can reach 806 kJ mol^−1^, a much larger negative potential than the thermodynamic value is needed in order to reduce CO_2_ [18]. The diversity of CO_2_ reduction products reduces the selectivity of catalytic reduction to target products [19, 20]. In addition, ECR needs the participation of free protons or proton donors (such as HCO^3−^) to break the C=O double bond, so there is always a highly competitive hydrogen evolution reaction (HER) in ECR [21]. As a result, the key to the effective utilization of ECR technology lies in the rational design and development of electrocatalysts with high activity, selectivity, and stability. In the early period of the ECR research, the selection of electrocatalysts is mainly focused on bulk metals, especially noble metals such as Pt and Au (~ US $50 g^−1^) [22]. However, the high cost, poor long-term stability, and low natural reserves of noble metals hinder their commercialization. Carbon-based nanomaterials (CBN) have become one of the main effective materials to replace noble metal catalysts due to the advantages of wide source of raw materials, controllable structure, good chemical stability, and electrical conductivity [23, 24]. Despite these advantages, there is still a large gap between the electrocatalytic performance of pure carbon nanomaterials and noble metals.

After Dai’s pioneering work on nitrogen-doped carbon nanotubes was published in 2009, high-performance CBN containing various defects have attracted the interest of many researchers [25]. The introduction of defects can affect the overall charge state of the carbon skeleton, thereby increasing the density and activity of potential active sites and improving the overall electrocatalytic performance of carbon nanomaterials [26, 27]. For example, edge and topological defects have been studied extensively in electrocatalytic reactions such as HER and oxygen reduction reaction (ORR) because of their different electrochemical and thermodynamic properties from the matrix material [28, 29]. Their potential for ECR has only recently been recognized. In addition, the doping of heteroatoms (N, B, P, etc.) [30] and the introduction of metal single atoms (Ni, Mn, Cu, etc.) [31–33] can change the electronic structure of carbon nanomaterials, increase the number of active sites and the activity of each active site, and stabilize the intermediate products of CO_2_ reduction better, so as to improve performances for ECR.

In this review, we systematically summarize defect engineering of carbon-based catalysts for ECR in terms of main characterization techniques, evaluation parameters and mechanisms of ECR, defect (intrinsic carbon defects, heteroatom doping defects, metal atomic sites) construction engineering on carbon-based catalysts. Then, we turn our focus to discuss the structure–activity relationship of ECR in depth. Finally, the current challenges and future development directions are proposed in order to provide some guidance for the development of ECR.

## Evaluation Parameters and Mechanism of ECR

With the development of ECR technology, related evaluation parameters and reduction mechanisms are also gradually being researched, improved and unified to meet the requirements for accurate analysis, evaluation and comparison of the performance of different electrocatalysts. In this section, we will briefly summarize the commonly used evaluation parameters and the different reaction pathways for C_1_ (carbon monoxide (CO), methane (CH_4_), formic acid (HCOOH), formaldehyde (HCHO) and methanol (CH_3_OH)) and multi-carbon products (C_2+_, ethanol (C_2_H_5_OH), ethane (C_2_H_6_), ethylene (C_2_H_4_), etc.).

### Evaluation Parameters for ECR

The key performance parameters used to evaluate the activity, selectivity and stability of ECR electrocatalysts are mainly onset potential, over potential, energy efficiency, Faraday efficiency, Tafel slope (current density), partial current density, and turnover frequency.

1. *Onset potential*: Onset potential refers to the potential at which the reaction begins to take place. The more positive the onset potential is, the more easily ECR reaction take place on the catalyst.
2. *Over potential*: Over potential is the difference between working potential and theoretical potential, which reflects the driving force of ECR.
3. *Energetic efficiency (EE)*: EE represents the overall energy utilization rate forming the target product.
4. *Faradaic efficiency (FE)*: FE represents the percentage of the charge consumed by the reaction to form the target product over the total charge transferred during the reaction. It can be calculated by Eq. 1 [34]:
  1 $${\text{FE}} = \frac{\alpha nF}{Q}$$
  where *α* is the number of electrons transferred per molecule of the target product (e.g., α = 2 for reduction of CO_2_–CO), n is the number of moles of the target product produced by the reaction, *F* is the Faraday constant (96,485 C mol^−1^), *Q* represents the charge passed during the entire reaction. The FE directly reflects the selectivity of the catalyst for ECR.
5. *Tafel slope*: The Tafel slope indicates the logarithm relationship between the overpotential and the current density, where the current density is obtained by dividing the total current by the geometric surface area of the working electrode. It is generally believed that the smaller the Tafel slope, the better the catalytic performance of the electrocatalyst.
6. *Partial current density*: Partial current density is the effective current density that drives the formation of the target product, but it is affected by many factors, such as electrolyte and electrochemical cell. Therefore, in order to compare the partial current density of different electrocatalysts, the experimental conditions should be defined first.
7. *Turnover frequency (TOF)*: Turnover frequency refers to the reaction turnover of each catalytic active site per unit time. It reflects the catalytic activity of the unit active site on the catalyst.

### Mechanism of ECR

In order to further understand the influence of defective carbon-based catalysts on ECR activity from experiments and theoretical calculations, the mechanism of ECR needs to be understood carefully. During the ECR reaction, the molecular bonds in CO_2_ and water to form oxygen and CO_2_ reduction products (C_1_, including CO, CH_4_, HCOOH, HCHO, CH_3_OH, and C_2+_ hydrocarbons).

**Fig. 1:**
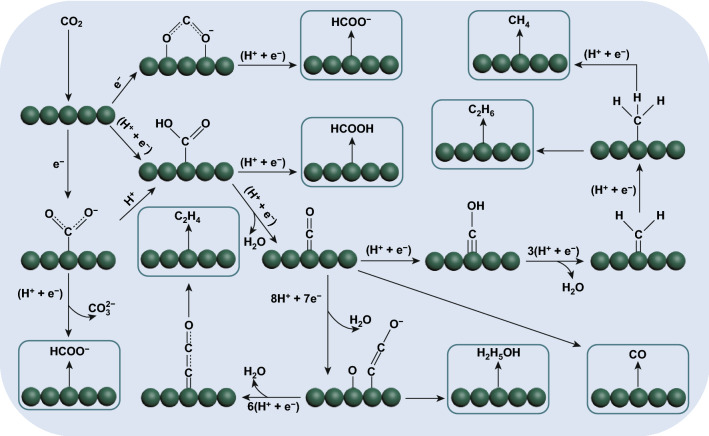
Potential ECR pathways to form the products of CO, HCOOH, CH_4_, C_2_H_4_, C_2_H_6_, and C_2_H_5_OH

Figure 1 shows the possible reaction pathway of ECR to common C_1_ and C_2+_ products. As we all know, the thermodynamics of CO_2_ is stable. In the actual process of ECR, it is necessary to apply a thermodynamic standard equilibrium potential far greater than that of CO_2_ reduction products to activate CO_2_ molecules. At the same time, the electrocatalytic reduction of CO_2_ is a multi-electron reaction process, including 2, 4, 6, 8, 12, or even more electron transfer reactions, which will form different products [21]. These reactions involving different electrons will compete with each other, as a result, the selectivity of the catalyst for reducing CO_2_ to the target product is reduced. In the aqueous reaction system, the thermodynamic standard equilibrium potential of various products produced by the reduction of CO_2_ is equivalent to the standard hydrogen evolution potential are as shown in Table 1 [35, 36]**.** Therefore, the HER will have a strong competition with ECR, which will further reduce the electrocatalytic activity of catalyst and selection of ECR.

**Table 1:** Main products of ECR and the corresponding standard potentials (The equilibrium potential is at 25 °C and 101.325 kPa, and with proton activity of 1 mol L^−1^)

Products	Equation	*E* (V vs SHE)
Formic acid	${\text{CO}}_{2} + 2{\text{H}}^{ + } + 2{\text{e}}^{ - } \to {\text{HCOOH}}$	− 0.61
Formate	${\text{CO}}_{2} + 2{\text{H}}_{2} {\text{O}} + 2{\text{e}}^{ - } \to {\text{HCOOH}}^{ - }$	− 1.49
Carbon monoxide	${\text{CO}}_{2} + 2{\text{H}}^{ + } + 2{\text{e}}^{ - } \to {\text{CO}} + {\text{H}}_{2} {\text{O}}$	− 0.52
Formaldehyde	${\text{CO}}_{2} + 4{\text{H}}^{ + } + 4{\text{e}}^{ - } \to {\text{HCHO}} + {\text{H}}_{2} {\text{O}}$	− 0.51
Methanol	${\text{CO}}_{2} + 6{\text{H}}^{ + } + 6{\text{e}}^{ - } \to {\text{CH}}_{{3}} {\text{OH}} + {\text{H}}_{2} {\text{O}}$	− 0.38
Methane	${\text{CO}}_{2} + 8{\text{H}}^{ + } + 8{\text{e}}^{ - } \to {\text{CH}}_{4} + 2{\text{H}}_{2} {\text{O}}$	− 0.24
Ethylene	$2{\text{CO}}_{2} + 12{\text{H}}^{ + } + 12{\text{e}}^{ - } \to {\text{C}}_{2} {\text{H}}_{4} + 4{\text{H}}_{2} {\text{O}}$	− 0.34
Hydrogen	$2{\text{H}}^{ + } + 2{\text{e}}^{ - } \to {\text{H}}_{2}$	− 0.42

In recent years, the FE of electrocatalytic selective reduction of CO_2_–CO has been greatly improved, even the FE_CO_ of some catalysts is close to 100%. A large number of theoretical and experimental studies have proved that the decisive step of ECR is that CO_2_ adsorbs an electron on the catalyst surface and is activated to form a radical anion *CO_2_^·−^, which is also the first step of ECR [19, 37–39]. The most common reaction mechanism for ECR–CO or HCOOH (formate (HCOO^−^)) is that when the C atom in *CO_2_^· −^ combines with the catalyst, a proton coupled electronic transfer to forms key intermediate *COOH, and then forms *CO through another proton coupled electronic transfer, and finally desorption to obtain CO. When the O atom in *CO_2_^·−^ combines with the catalyst, O atom will be protonated to form *OCHO, and then through proton coupled electron transfer to form HCOOH or formate [40].

With the developed of design for ECR catalysts, recent studies have begun to reduce CO_2_ into CH_4_, CH_3_OH, HCHO, and high-density C_2+_ products, and some research progress has been made in the reaction mechanism of ECR into these products. For the mechanism of ECR to CH_4_, CH_3_OH, and HCHO, theoretical and experimental studies show that *CO is the main intermediate of CO_2_ reduction [19, 41]. *CO can be hydrogenated to *HCO, *H_2_CO and *H_3_CO. Among them, *H_2_CO intermediate can be desorbed to HCHO, and *H_3_CO intermediate can be reduced to CH_3_OH. There are currently two main reaction pathways from *CO to CH_4_. One is the route proposed by Nie et al. which can be reduced to *C by *COH intermediate to generate CH_4_ [42]. That is to say, *CH, *CH_2_, and *CH_3_ are further reduced by *C, and finally CH_4_ is formed. This conjecture was later proved to be feasible in the reduction calculation of CO_2_ on the copper supported polymeric carbon nitride catalyst [43]. The other is *CO → *CHO → *CHOH → *CH → *CH_2_ → *CH_3_ → *^+^CH_4_, which was proved by Lu's et al. [44] through theoretical and experimental combination that this is the most supported path for reduction to CH_4_ at − 1.0 V versus standard hydrogen electrode (SHE). In the mechanism of CO_2_ reduction to C_2+_ products, it is believed that the subsequent reduction reaction of *CO intermediate determines which kind of C_2+_ product the catalyst electrocatalysts CO_2_ to finally reduce [45]. For example, C_2_H_6_ is *CO through proton-coupled electron transfer to form *CH and then protonation to form *CH_3_. Finally, the dimerization of *CH_3_ forms C_2_H_6_, while CH_3_COOH/CH_3_COO^−^ is formed by CO insertion into *CH_2_ [46].

Based on the in-depth understanding of the mechanism of ECR, low-cost electrocatalysts with excellent catalytic activity, high selectivity, and long durability are required to realize the large-scale application of ECR technology. It is considered that Au [47], Ag [48], Zn [49], and Pd [50] are more strongly bound to the intermediate *COOH, and tend to selectively form CO. At the same time, some transition metals such as Cd, Hg, In, Sn, Pb, and Bi are considered as selective catalysts for the formation of formic acid (formate) [51, 52]. Cu-based catalysts are used to reduce CO_2_–C_2+_ products. However, these catalysts used in ECR have some common disadvantages, such as low selectivity and poor durability, which hinder their wide application. Carbon-based catalysts have become one of the most promising materials to replace metal materials for ECR due to the advantages of wide source of raw materials, controllable structure, good chemical stability, and electrical conductivity, and has been widely concerned and studied in recent years [23, 24]. Among them, defective CBN have generally better ECR activity after design, and have become a research hotspot for improving ECR performance.

## Defect Construction Engineering on Carbon-Based Catalysts

**Fig. 2:**
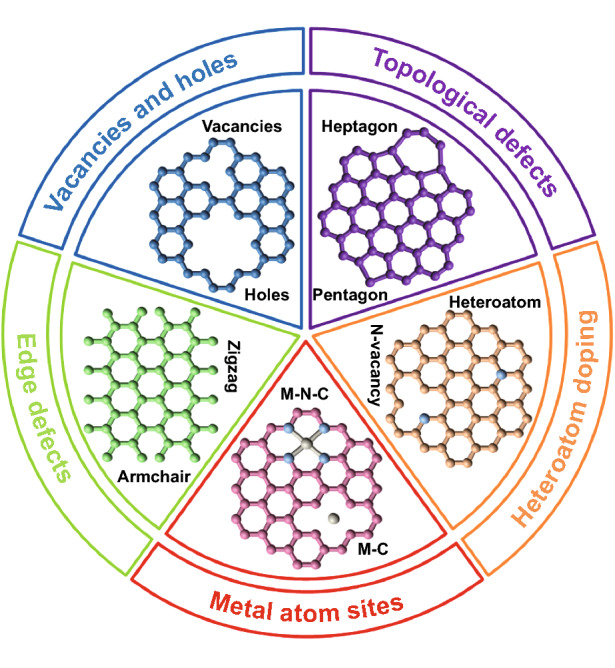
Schematic illustration of the type of defective CBN

According to the second law of thermodynamics, defects in crystalline materials cannot be eliminated, which also applies to CBN [24]. The research on the construction engineering of defective CBN focuses on point defects, which can be divided into intrinsic carbon defects (it is formed by thermal vibration of lattice atoms without any dopants, mainly including edges, vacancies, holes or topological defects), and extrinsic defects (it is mainly caused by heteroatom or metallic atomically dispersed active sites) (Fig. 2) [24, 53].

### Construction Engineering of Intrinsic Defects

The intrinsic carbon defects are mainly edge and topological defects. Edge defects, which make the material edge full of a large number of unpaired π electrons, can effectively accelerate the transfer of electrons and reduce the formation energy of key intermediates. At the same time, the edge carbon atoms show higher charge density and can be used as active sites [54]. The edges of carbon materials with hexagonal network structure can be divided into zigzag and armchair edges. The mechanical ball milling method has been proved to have the universality of making graphite carbon skeleton produce more edge defects [55]. It can effectively reduce the size of carbon nanomaterials and introduce more exposed edge sites into the carbon skeleton. For example, song et al. prepared a new type of defect rich graphene block (DGB) with controllable defect density and high filling density by a simple ball milling process with expanded graphene (EG) as the precursor [56]. In the process of ball milling, the high-energy mechanical impact force introduces a large number of intrinsic defects on graphene block. Chemical oxidation or etching is also an effective method to obtain carbon nanomaterials rich in edge defects. Shui et al. [57] used concentrated sulfuric acid and potassium permanganate to break the C–C bond and partially sheared the multi-walled carbon nanotubes, which were then reduced at high temperature under an argon atmosphere to obtain zigzag-edged graphene nanoribbons with a carbon nanotube backbone (GNR@ CNT). The zigzag-edged GNR@ CNT catalyst has excellent activity and stability, even better than carbon materials with doping defects. Plasma etching can quickly produce defects on the surface of the material without destroying the nanostructure [58]. Dai et al. [59] modified the carbon material with argon radio frequency plasma to obtain a dopant-free electrocatalyst rich in intrinsic defects. TEM characterization showed a large number of holes and edge defects on the graphene.

Topological defects, the introduction of topological defects will interfere with the electronic symmetry of aromatic rings, causing local charge redistribution, and the adjacent carbon atoms can be optimized to become the active sites of the electrocatalyst [60–62]. Topological defects usually exist in the carbon skeleton as non-hexagonal structures such as pentagons, heptagons, and octagons [63]. They are often constructed by in-situ etching, chemical vapor deposition (CVD) and nitrogen removal. Mu et al. [60] used fullerene as the framework and KOH as the etchant to preserve and expose the intrinsic carbon pentagons by in-situ etching. The presence of intrinsic pentagons defect structure in the catalyst was observed by aberration-corrected scanning transmission electron microscopy (AC-STEM). Nitrogen removal is the process of carbon-based nanomaterials in the high-temperature treatment process, the original N atoms are removed, forming edge or vacancy defects in the carbon skeleton. The resulting dangling bonds of some carbon atoms will be connected with each other, further forming topological defects [64]. This method can also limit the intense thermal motion of C atoms to avoid the recovery of the defective carbon skeleton formed. For example, Chen et al. completely removed pyridinic-N and pyrrolic-N in N-rich porous carbon particles by NH_3_-heat treatment to obtain a high-density topologically defective carbon catalyst [65]. X-ray absorption near-edge structure (XANES) measurements confirmed that as the processing temperature increases, the N content decreases and the topological defect density continues to increase, and the catalytic performance of CO_2_ reduction was also improved.

### Construction Engineering of Heteroatom Doping

In addition to the effect of intrinsic defects on the ECR catalyst, heteroatom doping (such as B, N, P, and F) with different electronegativity into the carbon network will introduce asymmetric charge, redistribute the spin density, break the electrical neutrality of carbon matrix, optimize the electronic properties of carbon materials to induce the generation of charged active sites [61, 66, 67]. N-doping is the most commonly used dopant in heteroatom doping. The main reason is that the pyridinic-N configuration has lone electron pairs, which can significantly enhance the binding of active sites on the catalyst surface with CO_2_ molecules and key reaction intermediates [68]. Compared with N-doping, B-doped CBN are relatively less studied, but B-doped CBN will also introduce asymmetric charge and spin density, so as to stabilize the key reaction intermediates and reduce the activation barrier of CO_2_ reduction. P doping can not only adjust the local electronic structure, but also cause greater distortion of the carbon lattice (because of the larger size of P), introducing more structural defects, and thus improving the catalytic performance of the electrocatalyst. Doping F into the carbon lattice will form a C–F bond with covalent or ionic or semi-ionic properties [69]. Because of the maximum electronegativity of F, the bonded carbon atoms will have high positive charge and become the active sites of ECR reaction, and the more positive charge the bonded carbon atoms have, the more active the C–F bond will be and the more favorable it will be for ECR [70].

Pyrolysis and CVD are 2 main methods for the construction of heteroatom doped carbon-based catalysts. Ajayan and co-workers synthesized N doped three-dimensional (3D) graphene foam (NG) by CVD method. The authors first use methane as the precursor and grow 3D graphene foam on the nickel foam template by CVD method, and then doped with nitrogen rich graphite-C_3_N_4_ at different temperatures to create N-defects. Finally, NG was prepared by hydrochloric acid etching nickel skeleton, repeated washing and freeze-drying [71]. Einaga et al. [72] used B(OCH_3_) as the B source to synthesize B-doped diamond (BDD) on Si(111) wafers by microwave plasma-assisted CVD, which has excellent stability for electrocatalytic reduction of CO_2_ to formaldehyde (> 20 h). Li et al. chose onion-like carbon (OLC) as the phosphorus-doped carbon host, and prepared phosphorus-doped OLC by CVD (P-OLC-CVD) [73]. Wang et al. [74] synthesized F-doped carbon (FC) catalyst by pyrolyzing a mixture of commercial BP 2000 and polytetrafluoroethylene (as F source). The element mappings of as-prepared FC catalyst showed that carbon and F distributed uniformly.

### Construction Engineering of Metallic Atomically Dispersed Active Sites

The introduction of metal atoms can improve the ECR performance of the catalyst due to its high dispersion and atom utilization [75]. Among them, metal-N–C (M-N-C) materials have attracted extensive attention and research after they were found to have high ECR performance in 2015 [76]. The main reasons for the excellent ECR performance of this material are as follows. (1) Metal atoms directly combine with C–N atoms in carbon network to form metallic atomically dispersed active sites [77]. (2) The size effect of metal single atoms gives CBN unique electronic structural characteristics and high specific surface area [78]. (3) The introduction of metal atoms changes the coordination environment of surface atoms, reduces the coordination number of surface atoms, decreases the over potential of the catalyst, increases the current density of the catalyst, and reduces the Gibbs free energy of the formation of key intermediates and the desorption energy of the final product in ECR reaction [35]. It is well known that the surface free energy of metal single atoms is large, and it is easy to migrate and agglomerate in the process of pyrolysis synthesis [79]. Therefore, various strategies are used to prevent agglomeration, such as strategies of spatial confinement [80], coordination design [81], and defect engineering [82]. Spatial confinement and coordinated design strategies usually use nitrogen atoms to confine isolated metal atoms. Li et al. [83] used the spatial confinement strategy to generate Ni single atoms distributed in nitrogen-doped porous carbon (Ni SAs/N–C) for ECR. The outstanding ECR performance of Ni SAs/N–C is mainly due to the existence of a large number of low coordination N_i_-N_x_ sites on its surface, which can be strongly combined with CO_2_^·−^. Wang et al. [84] used bimetallic Co/Zn zeolitic imidazolate frameworks as precursors to synthesize monoatomic Co-N_x_ catalysts. The N coordination number around the Co atom can be varied by controlling the pyrolysis temperature. The low coordination number of Co-N_2_ improves the ECR performance of the catalyst. Jiang and co-workers synthesized a new type of monodisperse Cu atoms are anchored on nanodiamond graphene with a large number of surface defects (Cu_1_/ND@G) using Cu(NO_3_)_2_·3H_2_O as the Cu precursor [85]. The strong interaction between the Cu atoms and the graphene substrate makes part of the positively charged isolated Cu atoms and C atoms form a three-coordinated environment, which is anchored at the defect site. It is precisely because of this unique structural feature that Cu_1_/ND@ G has high activity and high selectivity.

By understanding the effect of specific defect sites on the catalysts, the activity sources of different defect sites in different electrocatalytic reactions can be clarified. At the same time, we also need to understand the relationship between each structural defect and the ECR performance of the catalyst, so as to provide a new idea for the defect engineering of carbon-based electrocatalysts, and prepare high-performance ECR catalysts to meet the requirements of industrial production. Therefore, in the next section we will focus on the structure-effect relationship of defective carbon-based catalysts used in ECR in recent years.

### Characterizations of Carbon Defects

In order to better understand the internal catalytic mechanism of different defect sites, more and more advanced characterization techniques have been developed in recent years to meet the requirements of detection and analysis of defect types in electrocatalytic reactions. These advanced characterization methods can generally be divided into direct observation (e.g., electron microscopy) and indirect analysis (e.g., spectral characterization and physical structural analysis).

Electron microscopy plays an important role in the direct imaging of catalysts, and its resolution is at the nanoscale and even atomic level [86]. Aberration correction in scanning transmission electron microscopy (STEM) and high-angle annular dark field scanning transmission electron microscopy (HAADF-STEM) are used to directly characterize the surface defects of the catalyst. Thereinto, HAADF-STEM is the most commonly used one. Compared with bright field images, dark field images are more conducive to the observation of defects. It can realize the observation of the honeycomb structure of carbon-based catalysts on the sub-nanometer or even atomic scale, so as to identify the defect type [87]. STEM can increase the resolution without increasing the acceleration voltage. It can be combined with energy-dispersive spectroscopy (EDS) or electron energy loss spectroscopy (EELS) to further achieve subangstrom resolution [24, 88].

Spectral characterization techniques for indirect analysis of catalyst defects mainly include Raman spectrum, X-ray photoelectron spectroscopy (XPS), and X-ray absorption spectroscopy (XAS). In Raman spectrum, the D band represents defects and disorder induction, and the G band signifies the graphitization characteristics of the sp^2^ network. The ratio of D band to G band (*I*_D_/*I*_G_) can be used to describe the degree of defects in the carbon structure [89]. For XPS, in addition to the C-*sp*^2^ bond, the existence of other fitted bonds (C–*sp*^3^, C–N, C–O, C=O) in the C1s spectrum can reflect the 3-dimensional carbon structure and rich intrinsic defects of the catalyst [60] X-ray absorption spectroscopy (XAS) can reveal the geometry of atoms and is an effective tool for characterizing defects. XAS usually includes X-ray absorption near-edge structure (XANES) and extended X-ray absorption fine structure (EXAFS). XANES usually correspond to the structures found near the adsorption boundary, which can provide valuable information about the oxidation state, binding environment and local geometry of the absorbed atoms. EXAFS refers to the oscillating part of the spectrum, which can provide information about the coordination number and chemical bond length around the adsorbed atom [67, 75]. As the physical structural analysis technique, N_2_ adsorption–desorption isotherms analyzes the Brunauer–Emmett–Teller (BET) specific surface area and pore size distribution of the catalyst. To a certain extent, it can reflect the differences of morphological and structural of different catalysts [90].

## Activity Origin of Defective Effect on ECR

### Activity Origin of Intrinsic Carbon Defects

**Fig. 3:**
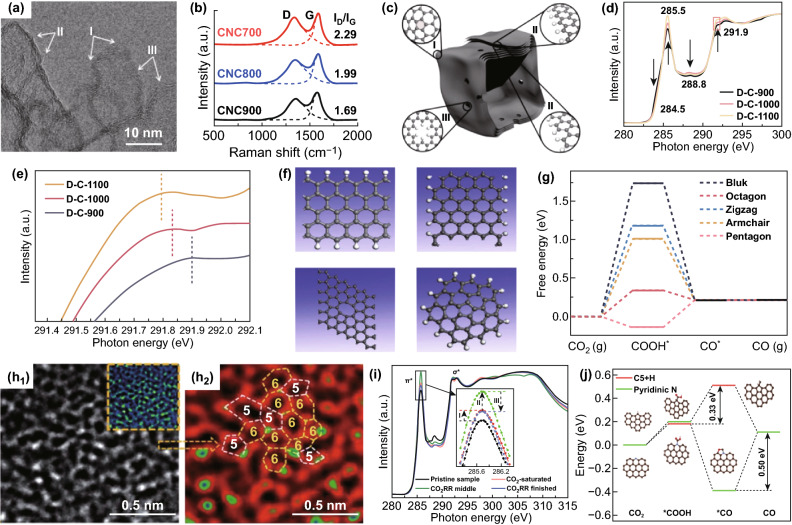
Intrinsic carbon defects. **a** HRTEM image of CNC700. **b** Raman spectra. **c** Schematic structure characters of the carbon nanocages [91]. Copyright © 2015, American Chemical Society. C K-edge NEXAFS spectra **d** and **e** expanded view for D-C-X. **f** Defect models used for theoretical calculations, **g** DFT calculations for ECR activities of different defects [92]. Copyright © 2019, John Wiley & Sons, Inc. Magnification of one segment of the HRTEM image **h1** and **h2** after fast Fourier transformation (FFT) filtering. **i** K-edge XAS curves of DHPC with different treatment modes. **j** Free energy diagram for CO2 reduction to CO over different defect sites [93]. Copyright © 2020, the Royal Society of Chemistry

With the deepening insights, it was found that the proper design of intrinsic defects in the carbon skeleton can affect the overall charge state of undoped carbon nanomaterials, increase the density of active sites, and thus improve electrocatalytic performance [27, 87]. A typical example is that Hu et al. synthesized undoped carbon nanocages (CNC) [91]. According to the characterization results, the sample obtained by pyrolysis at 700 °C have the highest specific surface area and the largest *I*_D_/*I*_G_ ratio, indicating that there are abundant defects in the corner, fringes and holes (Fig. 3a–c), so that the samples have the best ORR activity. Pure carbon nanomaterials with intrinsic defects have been applied to the electrocatalyst research of ECR in recent 2 years. More recently, Zhang et al. [92] reported that intrinsic carbon defects improve the catalytic activity of ECR. They synthesized a series of N-doped carbon spheres with defects for ECR test, and found that the ECR performance of the catalyst was positively related to the defect concentration, but negatively related to the N content, which is similar to that proposed by Yao’s et al. [29]. In order to verify the hypothesis that the intrinsic carbon defects are the active sites of ECR, a series of carbon catalysts without heteroatom doping were prepared. The NEXAFS spectra show that the defects of *sp*^2^ (octagonal and pentagonal) rather than the edges of *sp*^3^ (armchair and zigzag) are positively related to the ECR activity of the defective porous carbon catalysts, and as the pyrolysis temperature increases, the defect content continues to increase, the better the activity and selectivity of the catalyst (Fig. 3d–f). The mechanism of carbon defect promoting ECR performance was also verified by DFT calculations. As shown in Fig. 3g, for perfect *sp*^2^ carbon, the free energy required for the formation of *COOH by armchair and zigzag edge defects increases significantly, while the free energy required for pentagonal defects decreases significantly. For octagonal defects, due to the optimization of *COOH adsorption, the partially positive C atoms promote the conduction of electrons, thereby further promoting the reduction of *COOH–*CO. It can be seen from Fig. 3g that *CO desorption to form CO is an exothermic process, indicating that the reaction can proceed spontaneously. Overall, the ECR activity of all defect types is better than perfect *sp*^2^ carbon (bulk graphene), with octagonal and pentagonal defects performing best. Kang et al. [93] synthesized a hierarchical porous carbon catalyst rich in intrinsic defects (DHPC) (Fig. 3h). The mesoporous and carbon defect structure in DHPC improved its adsorption and activation capacity for CO_2_. FE_CO_ can reach 99.5% at − 0.5 V versus RHE in 0.5 M KHCO_3_ electrolyte. The DFT calculation shows that pentagon defects can adsorb CO_2_ spontaneously, and the barrier of DHPC electrocatalytic reduction of CO_2_–CO is lower than that of pyridine-n-doped catalyst, but it still reaches 0.33 eV. The reason may be due to the strong adsorption of *COOH radical by zigzag edge site and the strong binding of *COOH and *CO by armchair edge (Fig. 3i, j). Moreover, desorption of intermediate *CO on DHPC catalyst is also a spontaneous process. This also verified the characterization results of XAS that the pentagon defects was the active center of electrocatalysis. The intrinsic carbon defect engineering is expected to become a new paradigm for improving the performance of ECR catalysts and provide guidance for improving and developing new carbon-based electrocatalysts. It can be seen from the existing reports that the application of intrinsic carbon defect catalyst in ECR is mainly to reduce CO_2_–CO. the topological defect is the main active site, which can spontaneously adsorb CO_2_, and the adsorption energy of intermediates is not too strong, which is conducive to the further reduction and desorption of intermediates.

The catalytic performance of undoped carbon nanomaterials can be greatly improved by properly adjusting the intrinsic defects of carbon skeleton, and intrinsic defects will inevitably exist in the synthesized carbon nanomaterials. Therefore, combining various advanced characterization techniques and theoretical calculations, studying the effect of intrinsic carbon defects on the ECR performance of the catalyst is of great significance to obtain a more efficient and stable catalyst.

### Activity Origin of Heteroatom Doping Defects

**Fig. 4:**
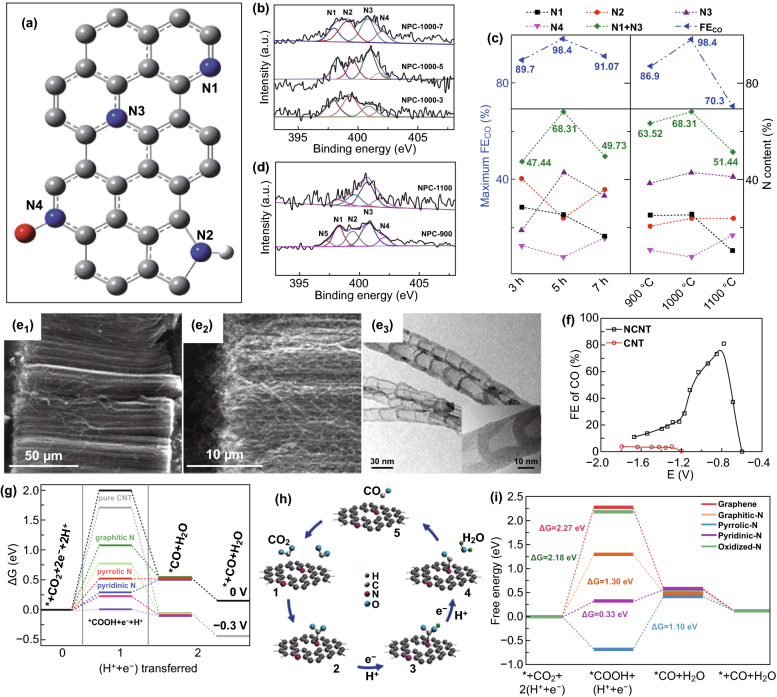
Heteroatom doping in carbon materials. **a** Schematic illustration of the structure of different N species in N-graphene sheet [105]. Copyright © 2012, the Royal Society of Chemistry. **b** N 1 s XPS spectra of NPC-1000-t. **c** Atomic contents of each N moiety, N1 + N3 and maximum FECO of NPC. **d** N 1 s XPS spectra of NPC synthesized at 900 and 1000 °C [106]. Copyright © 2020, John Wiley & Sons, Inc. **e1**, **e2** SEM images of NCNTs, **e3** TEM images of NCNTs (inset shows a single multiwall NCNT). **g** Free energy diagram for ECR–CO on CNTs and NCNTs [107]. Copyright © 2015, American Chemical Society. **h** Illustration of the ECR processes on N-GRW catalysts. **i** Free energy diagram of ECR on various N-GRW catalysts [66]. Copyright © 2018, John Wiley & Sons, Inc

Incorporation of heteroatoms with different electronegativity in the carbon skeleton will break the periodic structure of the original carbon, and the heteroatoms will replace part of the carbon atoms into the *sp*^2^ hybridized network [94, 95]. Heteroatom doping can optimize the electronic structure of carbon materials, redistribute the spin and charge density locally, and improve the surface adsorption/desorption behavior of intermediates [96–99]. In addition, the doping of heteroatoms can also stimulate the adjacent carbon atoms to improve the conductivity of carbon materials, thus enhancing the overall electrocatalytic activity of carbon materials [100]. The heteroatoms incorporated into carbon-based materials to construct defects are generally B [101], N [102], O [103], F [70], P [73], and S [104]. Among them, N atom is the most commonly used doped heteroatom because of its smaller atomic radius and larger electronegativity than carbon atom. N-doping in carbon nanomaterials generally forms four N configurations including pyridinic, pyrrolic, graphitic, and oxidized N (Fig. 4a), which will have different effects on the catalytic activity [105]. Most studies revealed that pyridinic-N is the main active center of ECR in these four N configurations because its lone electron pairs [67]. For example, Ajayan et al. [71] reported that N-doped 3D graphene foam structure was used for ECR–CO. This structure has a very low onset overpotential (− 0.19 V) and has an activity superior to that of noble metals. The maximum FE of CO is 85% at overpotential of − 0.47 V RHE and good stability for at least 5 h. DFT investigation revealed that pyridinic-N can reduce the formation of adsorbed *COOH free energy barrier and was the most active site for ECR–CO. However, due to their complex coexistence, it is challenging to distinguish which N configuration is the real active center. Huang et al. [106] synthesized N-doped porous carbon (NPC) electrocatalysts with rich pore structure and high concentration of active N (pyridinic and graphitic N). The obtained NPC showed excellent ECR activity with an onset potential of − 0.35 V and *FE*_CO_ of 98.4% at − 0.55 V versus RHE, which is one of the highest reported values of NPC-based ECR electrocatalysts. NPC catalysts containing different N species including pyridinic (N1), pyrrolic (N2), graphitic (N3), and oxidized (N4) were obtained by controlling the calcination time and temperature. The N1 + N3 contents in the samples calcined at 1000 °C for 5 h reached the maximum value of 68.31%, and the best FE of ECR to CO was similar to that of N1 + N3, as shown in Fig. 4b–d. This demonstates that N1 and N3 in the obtained NPC are the active sites of ECR. DFT calculation also proved this point, but it is worth noting that the formation of *COOH intermediates for pyrrolic-N is a spontaneous process. However, due to the strong adsorption of pyrrolic-N–*COOH, it is necessary to overcome the large energy barrier for further reduction to *CO, so pyrrolic-N cannot be the active site to improve the catalytic performance of NPC catalyst. Ajayan et al. [107] prepared N-doped carbon nanotubes (NCNTs) with high activity and selectivity (Fig. 4e). When ECR testing was performed in 0.1 M KHCO_3_, NCNTs exhibited lower overpotential (− 0.18 V) and higher FE for selective reduction to CO products (80% at − 0.78 V versus SHE) (Fig. 4f). Theoretical calculations indicate that the potential-limiting step of NCNTs catalyst ECR is the formation of absorbed *COOH intermediate. And the free energy of pyridinic-N to form *COOH intermediate is lower than that of pyrrolic-N and graphitic-N (Fig. 4g). Moreover, *COOH can be further spontaneously reduced to *CO intermediate on pyridinic-N, and can be easily desorbed to form CO. These results indicate that the most effective N configuration is pyridinic-N. A similar result was also reported by Liu et al. that N-doped graphene nanoribbon networks (N-GRW) catalyst exhibits superior ECR activity [66]. The FE of reducing CO_2_ to CO products at an over potential of 0.49 V (versus RHE) reaches 87.6%. Based on XPS characterization and Gibbs free energy calculation, it is proved that pyridinic-N is the more active sites for CO_2_ adsorption, *COOH intermediate formation, and *CO desorption during ECR (Fig. 4h, i). Different carbon matrix materials also affect the catalytic performance of heteroatom doped carbon-based catalysts. Due to their small size, carbon quantum dots often bring unexpected opportunities for materials. For example, Ajayan et al. [108] synthesized N-doped graphene quantum dots (NGQDs) electrocatalyst for ECR. The NGQDs catalyst not only has low over potential and high reduction current density, the selectivity and yield of CO_2_ reduction to ethylene and ethanol are comparable to copper-based electrocatalysts. Nanodiamond is mainly composed of *sp*^3^ carbon. Although the conductivity of pristine nanodiamond is low, its conductivity can be significantly improved after doping with heteroatoms, which brings attractive electrochemical performance. In particular, N-doped nanodiamond has a higher HER over potential than most reported electrocatalysts [109], which is very favorable for ECR. Quan et al. prepared N-doped nanodiamonds (NDD), which overcomes the common problem of low selectivity of the catalyst in reducing CO_2_–C_2+_. NDD catalyst can preferentially and efficiently reduce CO_2_ to acetate, and the FE of acetate can reach 91.2–91.8% at − 0.8 to − 1.0 V versus RHE [110]. Through comparative experiments, the author also found that N-*sp*^3^C can electrocatalyze CO_2_ reduction more effectively than N-*sp*^2^C due to its better activity. The adsorption and desorption of CO_2_ and reduction intermediates by doped heteroatoms is explained by taking N heteroatoms as an example. When N is doped into carbon nanomaterials, defect sites can be induced, and doped-N atoms can polarize adjacent carbon atoms. The generated positively charged carbon atoms and defects can promote the adsorption of CO_2_ on the catalytic surface and stabilize the generated *CO2^·−^ through electronic interaction, thereby lowering the energy barrier, promoting CO_2_ reduction and desorption of intermediates.

**Fig. 5:**
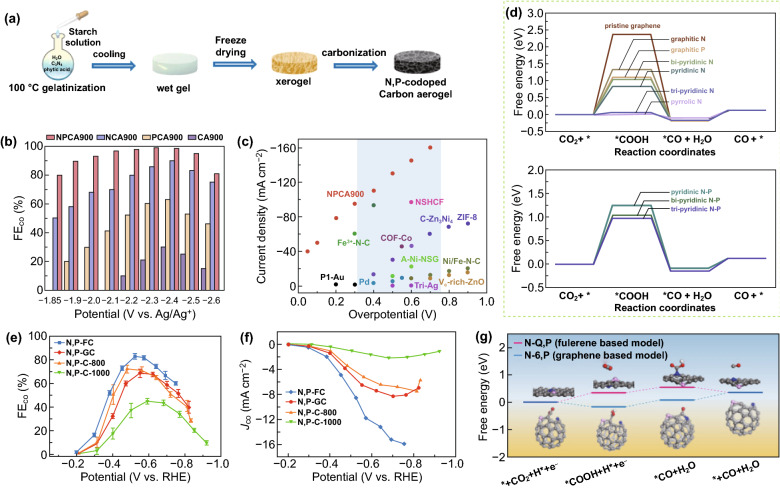
Heteroatom doping in carbon materials. **a** Schematic illustration of NPCA synthesis. **b** FE_CO_ for various catalysts. **c** Current density over NPCA900 compared with other catalysts. **d** Gibbs free energy diagrams for ECR–CO over simulated N- or P-doped carbon configuration [111]. Copyright © 2020, John Wiley & Sons, Inc. **e** FECO for various catalysts. **f** Partial current densities of CO on various catalysts. **g** Free energy diagram of ECR reaction to CO on various catalysts [95]. Copyright © 2019, The Royal Society of Chemistry

The diatomic co-doping may bring about the catalytic activity that the single heteroatom doped carbon material does not have because of the synergistic effect. Han et al. prepared N, P-co-doped carbon aerogels (NPCA) catalysts (Fig. 5a) [111]. The NPCAs achieves high FE while achieving high current density. When the NPCAs for ECR to CO at − 2.4 V versus Ag/Ag^+^, the *FE*_CO_ reaches 99.1%, and the partial current density is − 143.6 mA cm^−2^. It also shows that NPCA has higher active area and total electron conductivity than N or P single-doped carbon aerogels, which can facilitate the conversion of electrons from CO_2_ to its radical anions or other key intermediates. Through further comparative study, it is found that the reason why the NPCA catalyst has high FE and current density is that pyridinic-N has a high activity for reducing CO_2_–CO and co-doping of P and N can significantly inhibit the HER (Fig. 5b–d). The ECR activity of electrocatalyst is also affected by the different atomic ratio of dopant and carbon-based nanomaterials. Zhang et al. prepared N, P-coordinated fullerene-like carbon (N, P-FC) and N, P-coordinated graphite-like carbon (N, P-GC) by soft-template pyrolysis method for ECR performance test [95]. Different pyrolysis temperatures make the samples have different P/N atomic ratios, and the catalytic activity of the samples increases with the increase of the P/N atomic ratio. The N, P-FC obtained by pyrolysis at 900 °C has the best ECR activity (Fig. 5e, f). At the same time, it is found and confirmed by calculation that fullerene structure is more beneficial to ECR reaction than graphene structure (Fig. 5g). Consequently, the above studies provide guidances and references for designing and synthesizing high efficiency, high selectivity, and low cost heteroatom-doped carbon materials to replace noble metal catalysts for efficient ECR.

The co-doping of two different elements in carbon materials provides a new opportunity for the application of electrocatalysts. However, the catalytic performance of heteroatom co-doped carbon-based catalysts is not absolutely better than single-doped carbon-based catalysts. Only by properly adjusting the precursor, doping type, doping ratio and spatial distribution configuration can the ECR performance of carbon-based catalysts be optimized.

### Activity Origin of Metallic Atomically Dispersed Active Sites

The introduction of metallic atomically dispersed active sites is another common and effective method to improve the electrocatalytic activity of CBN [112, 113]. In recent years, metal single-atom electrocatalysts have been favored by researchers because of their extremely high atomic utilization rate [114–116]. These metal single-atom active sites are also a kind of carbon defect, because in the carbon network, metal atoms will directly bond with C–N atoms. This single-atom catalyst has high catalytic activity and excellent stability, and is widely used in various electrocatalytic reactions. Among them, the metal–nitrogen–carbon (M–N–C) catalyst not only has extremely high atom utilization rate, but also can reduce the chemical adsorption of hydrogen on the monoatomic metal coordinated with nitrogen (M-N_x_). While inhibiting the unwanted HER, the efficiency and selectivity of ECR are greatly improved [117–119].

**Fig. 6:**
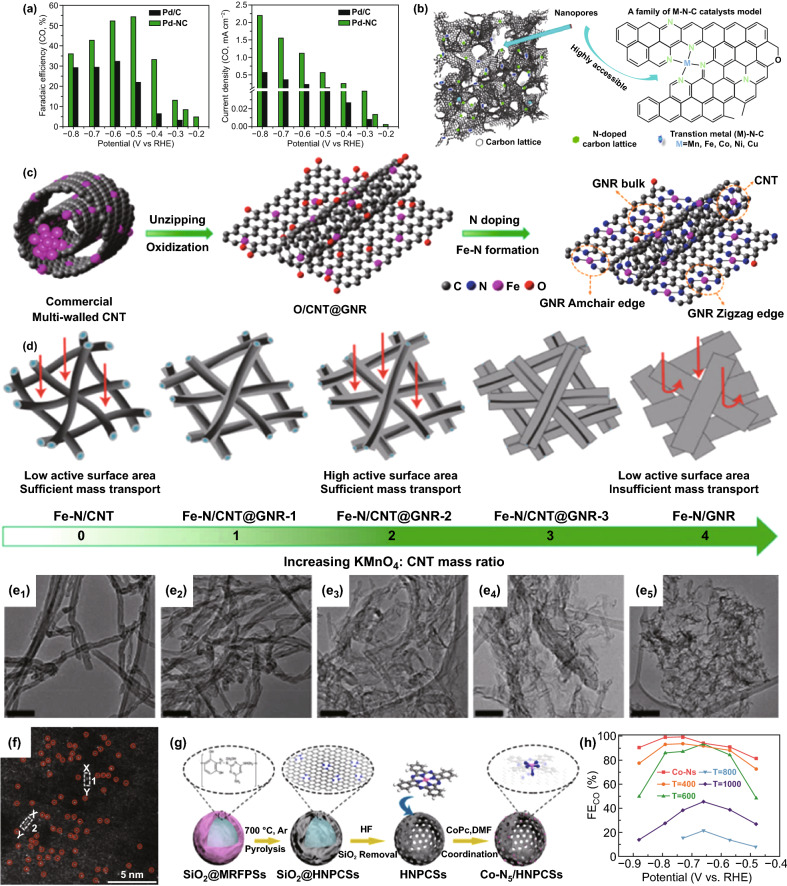
Metallic atomically dispersed active sites. **a** ECR performance of two catalysts [50]. Copyright © 2020, John Wiley & Sons, Inc. **b** Materials model and a schematic local structure [120]. Copyright © 2017, Springer Nature. **c** Schematic illustration of the formation of Fe–N/CNT@GNR. **d** Structural evolution GNR. **e** TEM images of samples obtained with different KMnO4: CNT mass ratios. Scale bar in e1–e5 is 100 nm [121]. Copyright © 2020, American Chemical Society. **f** HAADF–STEM image of Co1/PCN. **g**, **h** Schematic illustration and FECO of Co-N5/HNPCSs-T [115]. Copyright © 2018, American Chemical Society

M–N-C catalyst has been widely used in the study of ORR for decades, but it was used to study ECR reactions was for the first time proposed by Strasser et al. [76] in 2015 that Fe–N-C, Mn-N–C and Fe,Mn-N–C have high ECR performance. The metals used to form M–N-C catalysts are mainly transition metals and individual noble-metals such as Fe, Co, Ni, Cu, and Pd. Kattel et al. [50] prepared a N-doped carbon-supported Pd single-atom catalyst (Pd-NC) with Pd-N_4_ sites. The well-dispersed Pd-N_4_ active centers in the catalyst help stabilize the adsorbed CO_2_ reduction intermediates, thereby enhancing the ECR ability at low overpotentials (Fig. 6a). However, due to the rarity and expensiveness of noble metals, low-cost transition single metal atom catalysts that can achieve the catalytic activity of noble metals are currently hot research topics. Fe–N-C, Co–N-C, and Ni–N-C are the most concerned catalysts for selective ECR–CO. Strasser et al. synthesized a series of transition metal-doped nanoporous carbon materials (M–N-C, M=Mn, Fe, Co, Ni, Cu) using the N-coordinated strategy (Fig. 6b) [120]. Because of the difference of the binding strength between Fe–N-C, Co–N-C, and Ni–N-C and the reaction intermediates, Fe–N-C and Co–N-C have lower onset overpotential than Ni–N-C, but the selectivity of reducing to CO is lower than Ni–N-C. Notably, the strong binding of Fe–N-C and Mn-N–C with the *CO intermediate makes it difficult for *CO to desorb to form CO, which makes it possible to further reduce to CH_4_. Li et al. by controlling the amount of KMnO_4_ added into the solution of oxidized carbon nanotubes and high-temperature heat treatment it with urea obtained the isolated Fe-N_4_ sites supported on hierarchical carbon nanotube (CNT) and graphene nanoribbon (GNR) network (Fe-N/CNT@ GNR) with different GNR content [121]. Figure 6c–e show the structural evolution of the sample during oxidation. It can be seen that by adjusting the amount of KMnO_4_, CNT can be decompressed into CNT@GNR, and finally GNR. Among them, Fe-N/CNT@GNR-2 has four positions to accommodate the monodisperse Fe-N_4_ sites, namely CNT, GNR bulk and edges (armchair and zigzag edges) (Fig. 6c). The CNT and zigzag sites can promote the activation of CO_2_ and the formation of *COOH intermediates. The zigzag and armchair edges can weaken the adsorption of *CO and make *CO easier to desorb. The largest active area and sufficient mass transfer improves the ECR performance of Fe-N/CNT@GNR-2. Compared with other catalysts, it has the most positive onset potential (− 0.3 V) and the highest FE_CO_ (98%), and largest CO partial current density (22.6 mA cm^−2^ at − 0.76 V vs RHE). Co-N-C research is relatively fragmented due to low CO selectivity. However, in the study of Chen et al. [115], the coupling of Co-N_4_ with the N dopant in the nearby carbon network can improve the ECR performance. The atomically dispersed Co-N_5_ active centers are fixed on polymer-derived hollow N-doped porous carbon spheres (Co-N_5_/HNPCSs) through the N-coordination strategy as shown in Fig. 6f, g. The results of Co K-edge XANES indicate that the valence of Co is between + 2 and + 3, and EXAFS fitting knows that the Co–N coordination number is 5. The additional N coordination comes from the N atom in nearby HNPCSs. Co-N_5_/HNPCSs showed high *FE*_CO_ (> 90%) in the range of − 0.57 to − 0.88 V versus RHE (Fig. 6h). DFT calculation indicated that the atomically dispersed Co-N_5_ site is the main active center of CO_2_ activation and the rapid formation of key reaction intermediate *COOH, and because the binding force of Co-N_5_ and *CO is relatively weak, *CO is easy to desorb into CO.

**Fig. 7:**
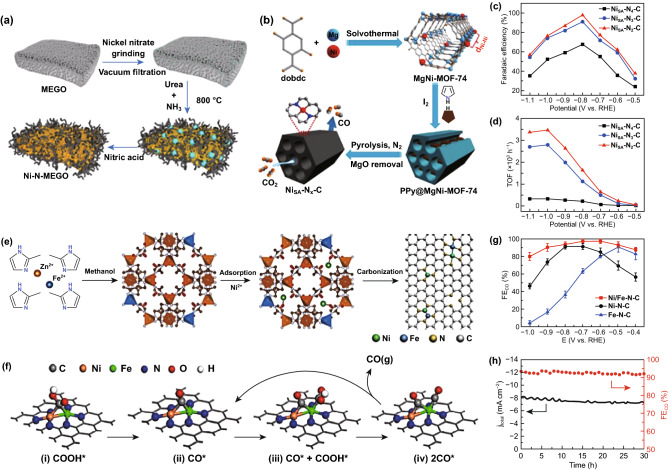
Metallic atomically dispersed active sites. **a** Scheme showing the synthesis of Ni–N-MEGO [117]. Copyright © 2019, Elsevier. **b** Schematic illustration of the ECR process of NiSA-Nx-C catalyst. **c** FECO for different catalysts. **d** TOFs of CO production over NiSA-Nx-C [122]. Copyright © 2020, John Wiley and Sons, Inc. **e** Schematic illustration of Ni/Fe–N-C synthesis. **f** Catalytic mechanism. **g** FECO of various catalysts. h Stability test for Ni/Fe–N-C at − 0.7 V [124]. Copyright © 2019, John Wiley & Sons, Inc

Compared to Fe–N-C and Co–N-C, Ni–N-C is the most popular M–N-C electrocatalyst due to its high activity and CO selectivity. Jiang et al. used microwave-peeled graphene oxide (MEGO) with high porosity and high surface defects as support, anchored the single-atom Ni on the MEGO support (Ni–N-MEGO) [117], making the loading amount of Ni atom reach 6.9% (Fig. 7a). The formation of the edge-anchoring coordinated unsaturated Ni–N active structure makes Ni–N-MEGO have better ECR performance. At an over potential of 0.59 V versus RHE, the partial current density and selectivity of CO reached 26.8 mA cm^−2^ and 92.1%. Theoretical calculations show that the edge-anchored unsaturated NiN_3_ and NiN_2_ (NH_2_) can adsorb CO_2_ and activate it to *COOH, and have the favorable energy for the desorption of *CO. The preparation and coordination environment control of single-atom catalysts are still a big challenge. Gong et al. constructed a host–guest cooperative protection strategy via introducing polypyrrole molecules in bimetallic MgNi-MOF-74 to synthesize single-atom (SA) Ni implanted N-doped carbon (NiSA-N_x_-C) catalysts [122], Ni–N coordination number is controlled between 4 and 2 (Fig. 7b). The low N coordination number of the single-atom Ni site in NiSA-N_x_-C is favorable for CO_2_ adsorption to form *COOH intermediates, so that the FE of CO reaches 98% and the turnover frequency reaches 1622 h^−1^ at − 0.8 V versus RHE (Fig. 7c, d). Bao et al. [116] also reached a similar conclusion. They synthesized Ni–N site-doped porous carbon catalysts with high loading and coordination unsaturated for ECR. The current density of the catalyst for ECR–CO can reach 71.5 ± 2.9 mA cm^−2^ at − 1.03 V versus RHE, and can maintain high FE_CO_ (92.0 ~ 98.0%) over a wide potential range (− 0.53 to − 1.03 V versus RHE). DFT results show that the lower the coordination number, the more conducive to the activation of CO_2_ and the improvement of ECR performance. For example, when the Ni–N coordination number is 2, it can not only adsorb CO_2_ molecules and has a low *COOH formation energy, but also can effectively inhibit the HER.

In recent years, the bimetallic-N–C catalyst has attracted extensive attention and research due to its bimetallic active center and possible synergy. Gong et al. [123] recently synthesized Zn and Co atoms coordinated on N doped carbon (Zn/Co–N–C) electrocatalysts for ECR. The catalyst has a high CO FE of 93.2% at − 0.5 V versus RHE during a 30 h test, and a higher CO partial current density of 71.6 mA cm^−2^ at − 0.8 V versus RHE. The author studied the influence of the neighborhood effect between monomers on the performance of ECR. XANES shows that there is an electronic effect between Zn/Co monomers. In situ-attenuated total reflection-infrared spectroscopy and DFT calculations reveal that the electronic effect between Zn and Co reduces the energy barrier for the formation of *COOH on the Zn site, thereby promoting the reduction of CO_2_–CO. Zhao et al. [124] using ZIF-8 as a template to successfully synthesized isolated diatomic Ni–Fe anchored nitrogenated carbon (Ni/Fe–N-C) catalysts (Fig. 7e). The large number of active centers exposed by the diatomic structure increases the activity of the catalyst. Ni/Fe–N-C has suitable adsorption/desorption energy for the intermediate products of ECR. In a wide potential range, CO_2_ can be reduced to CO with high selectivity, FE reaches a maximum of 98%, and has a robust stability (> 30 h) at − 0.7 V versus RHE (Fig. 7g, h). DFT calculation shows that the binding strengths of *COOH and *CO on CO-adsorbed Ni/Fe–N-C are weaker compared with bare Ni/Fe–N-C, therefore, after CO adsorption, the theoretical overpotential of Ni/Fe–N–C decreases and the potential barrier of catalytic formation of CO is greatly reduced. Based on this, the author proposes a post adsorption mechanism. As shown in Fig. 7f, the Fe–Ni diatomic sites are first passivated by strongly bounded *CO (i-ii) and then the reduction of CO_2_ occurs on the Fe sites (ii-iv), this mechanism is similar to that previously observed in HO-Co-N_2_ [125] and Ir-Co [126], which is very conducive to the ECR.

The atomically dispersed metal atoms on the carbon carrier generally act as the active center themselves or coordinate with the surrounding C atoms and heteroatoms to become the catalytic active center, which promotes the adsorption and activation of CO_2_ and reduces the potential barrier and desorption energy of key intermediates. However, the catalyst design is needed to achieve high selectivity reduction to specific products. Although bimetallic active center often bring unexpected catalytic performance to the catalyst due to the synergistic effect, the excellent catalytic performance of the catalyst is not only related to single and double doping, but also affected by many other factors, such as material morphology, specific surface area, active sites content and doping ratio. Therefore, it cannot be arbitrarily considered that the ECR performance of catalysts with diatomic active sites must be better than those with single-atom active sites.

Through constructing appropriate carbon precursor materials, atomic-level metals can be captured and stabilized. Due to the great difference of electronegativity between metal and carbon atom, sufficient charge transfer can be generated to make this kind of structural unit (M–N-C/M-C) become the active centers of electrocatalysis and reduce the activation energy barrier and over potential of the reaction. These strategies provide an important guidance for the preparation of high-performance ECR catalysts and are also one of the current research hotspots.

## Conclusions and Perspectives

Defect engineering design is an effective means to change the physical and chemical state of the carbon skeleton surface and improve its ECR performance. Specifically, the existence of intrinsic defects, the doping of heteroatoms and the introduction of metal atoms mainly change the local electronic structure of the carbon matrix to make the charge distribution unbalanced, so that the redistribution of the charge is used to optimize the carbon matrix, and increase the active sites density and catalytic activity of the catalyst. This paper summarizes main characterization techniques, evaluation parameters and mechanism of ECR, structure of defects and their effects on ECR electrocatalyst, based on the research progress in recent years, a detailed review of the sources and catalytic mechanisms of catalytic active centers with different defect carbon materials. And the ECR performance of different defective carbon-based catalysts is summarized in Table 2. However, despite the exciting progress, there are still many problems in the existing electrocatalyst and ECR system, which must be solved before the commercialization of ECR technology.

1. Control synthesis. It is very difficult to accurately introduce specific defects into CBN. Although the edge defects can be accurately synthesized by ball milling, etching and other methods, other defects cannot be accurately fabricated. In particular, the high-temperature pyrolysis process commonly used to prepare carbon-based electrocatalysts often causes bond rupture and reconnection, which will have a more or less influence on the activity of the electrocatalyst. Therefore, in order to determine the specific effects of specific defects on different electrocatalytic reactions, more controllable and accurate synthesis methods need to be developed.
2. Improve the selectivity of multi-carbon products (C_2+_). Multi-carbon oxygenates and hydrocarbon products with higher energy density are chemicals that are urgently needed in many industrial applications. However, most carbon-based catalysts are currently used to catalyze the reduction of CO_2_–C_1_ products such as CO and formic acid, but the selectivity to C_2+_ products is generally low, which is far from the ideal goal of practical application. The development of defective carbon-based catalysts with high activity and selectivity for the formation of C_2+_ products is critical, but also very challenging. To achieve this goal, further developments are needed in the synthesis method and understanding of the CO_2_ reduction mechanism of defective carbon-based catalysts.
3. Defect characterization. In general, the restructuring of the active site may occur during the reduction reaction, and the traditional in situ characterization method cannot reflect this structural change. Therefore, in situ/operando measurements have become an effective technique for deeply understanding the role of defects in electrocatalytic reactions, which can guide researchers to effectively design and protect active sites.
4. Large-scale synthesis of defective electrocatalysts for ECR. It is well known that the presence of defects in the catalyst can improve its electrocatalytic performance. However, since the formation of defects in the catalyst is a high-energy process, the defect content is extremely low, which can currently only be prepared in the laboratory. To meet the economic needs of the practical application of defective electrocatalysts in ECR, an efficient strategy for large-scale synthesis of defective electrocatalysts is urgently needed.
5. Mechanism analysis. The nature of the catalytically active sites in CBN and their detailed reaction pathways are unclear and need further clarification. Advanced simulation calculation technology can help to simplify the research process, overcome the complexity, and promote the understanding of the active sites, structure–activity relationship and reaction mechanism to a certain extent. Through the mutual verification of theoretical calculations and experimental results, the role and catalytic mechanism of the actual active center can be accurately determined.
6. Optimize ECR equipment or system. According to some techno economic analysis and prediction, ECR technology needs current density > 200 mA cm^−2^ to reach commercial level. The traditional H-cells obviously cannot meet this requirement. Therefore, we must optimize the ECR performance of CBN at the equipment or system level. For example, using flow cells, zero gap cells, or microfluidic electronic cells can increase the current density. Unfortunately, it is rarely used in ECR of carbon-based materials.

**Table 2:** Performance comparison of different carbon-based catalysts for ECR

Material	Reactor	Electrolyte	Product and FE (%)	Durability (h)	Current density (mA cm^−2^)	*E* versus RHE (V)	Refs
F-CPC	H-cell	0.5 M KHCO_3_	CO@88.30	12	37.5	− 1.0	[39]
Pd-NC	H-cell	0.5 M NaHCO3	CO@55	–	*j*_CO _~ 0.244	− 0.5	[50]
DPC-NH_3_-950	H-cell	0.1 M KHCO3	CO@95.2	24	2.84	− 0.6	[65]
N-GRW	H-cell	0.5 m KHCO3	CO@87.6	10	~ 0.029	− 0.49	[66]
NCNTs-ACN-850	H-cell	0.1 M KHCO3	CO@80	–	*j*_CO _~ − 3.5	− 1.05	[68]
FC	H-cell	0.1 M KHCO3	CO@93.1	–	0.394	− 1.22	[74]
P-OLC	H-cell	0.5 M NaHCO_3_	CO@81	27	*j*_CO _~ 4.9	− 0.90	[73]
Ni SAs/N–C	H-cell	0.5 M KHCO3	CO@71.9	60	10.48	− 1.0	[83]
D-NC-1100	H-cell	0.1 M KHCO_3_	CO@94.5	> 10	*j*_CO _~ − 1.0	− 0.60	[92]
DHPC	H-cell	0.5 M KHCO3	CO@99.5	–	*j*_CO _~ − 5	− 0.5	[93]
N,P-FC	H-cell	0.5 M NaHCO3	CO@83.3	–	~ 24	− 0.8	[95]
NG-800	H-cell	0.1 M KHCO3	CO@ ~ 85	5	*j*_CO _~ − 1.8	− 0.58	[71]
NPC-1000	H-cel	0.5 M KHCO3	CO@98.4	18	3.01	− 0.55	[106]
NCNTs	Flow-cell	0.1 M KHCO3	CO@80	10	*j*_CO _~ − 1.0	− 0.78	[107]
NGQDs	Flow-cell	1 M KOH	90	–	–	− 0.75	[108]
			C2H4@31			− 0.75	
			CH4@15			− 0.86	
			C2H5OH@11.8			− 0.74	
NDD	H-cell	0.5 M NaHCO3	Acetate@91.8	3	~ 0.8	− 1.0	[110]
NPCA	H-cell	0.5 M KHCO_3_	CO@99.10	24	*j*_CO _~ − 143.6	− 2.3 (vs Ag/AgCl)	[111]
Co-N_5_/HNPCSs	H-cell	0.2 M NaHCO3	CO@99.4	10	6.2	− 0.79	[115]
Co-N_2_	H-cell	0.5 M KHCO3	CO@94	–	18.1	− 0.63	[119]
Fe–N-C							
Ni–N-C	H-cell	0.1 M KHCO3	CO@65	–	–	− 0.55	[120]
			CO@85			− 0.78	
Fe- N/CNT@GNR	H-cell	0.5 M KHCO3	CO@96	5	23.8	− 0.76	[121]
Ni–N-MEGO	H-cell	0.5 M KHCO3	CO@92.1	21	*j*_*CO* _~ 26.8	− 0.7	[117]
NiSA-N_2_-C	H-cell	0.5 M KHCO3	CO@98	10	–	− 0.8	[122]
C-Zn_1_Ni_4_ ZIF-8	H-cell	0.5 M KHCO3	CO@98.0	–	22.0	− 1.03	[116]
Zn/Co–N-C	Flow-cell	0.5 M KHCO_3_	CO@93.20	30	26	− 0.50	[123]
Ni/Fe-NC	H-cell	0.5 M KHCO_3_	CO@98	> 30	9.5	− 0.70	[124]

In summary, ECR provides us with a good way to solve a series of environmental problems caused by greenhouse gases, and provides a good way to end man-made carbon cycle and store renewable energy. Although there are still many deficiencies in the current CO_2_ reduction technology, the defective carbon-based catalysts have unparalleled advantages over non noble metal catalysts and are very expected to replace noble metal catalysts. And a more rational design for the defect engineering of other materials (metal oxides and perovskite) with advanced technology will eventually realize the commercial application of CO_2_ reduction technology.
